# Prevalence of extended spectrum beta lactamase and plasmid mediated quinolone resistant genes in strains of *Klebsiella pneumonia, Morganella morganii, Leclercia adecarboxylata* and *Citrobacter freundii* isolated from poultry in South Western Nigeria

**DOI:** 10.7717/peerj.5053

**Published:** 2018-06-22

**Authors:** Olajumoke R. Akinbami, Samson Olofinsae, Funmilola A. Ayeni

**Affiliations:** Department of Pharmaceutical Microbiology, Faculty of Pharmacy, University of Ibadan, Ibadan, Nigeria

**Keywords:** Resistance genes, Chicken, Susceptibility, Antibiotics, Pathogens

## Abstract

A serious concern is arising on the coexistence of extended-spectrum beta-lactamase (ESBL) and plasmid mediated quinolone resistance (PMQR) producing bacteria in animal husbandry, which could be transferred to humans, especially in strains that may not be routinely screened for resistance. This study therefore tested the prevalence of ESBL and PMQR genes in selected bacteria isolated from poultry faeces. Faecal droppings of birds were collected from 11 farms in five states in South Western Nigeria. Bacteria were isolated from the samples on cefotaxime supplemented plates and identified with MALDI-TOF. The MIC was determined using VITEK system and resistance genes were detected with PCR. A total of 350 strains were isolated from different samples and selected strains were identified as 23 *Klebsiella pneumonia,* 12 *Morganella morganii,* seven *Leclercia adecarboxylata* and one *Citrobacter freundii.* All the species were resistant to gentamycin, trimethoprim/sulphamethaxole, tobramycin, piperacillin, cefotaxime and aztreonam (except *Morganella morganii* strains which were mostly susceptible to aztreonam). All the tested strains were susceptible to imipenem, meropenem and amikacin. All *Leclercia adecarboxylata* strains were resistant to ceftazidime, cefepime and fosfomycin while all* Morganella morganii* strains were resistant to fosfomycin, moxifloxacin and ciprofloxacin. All tested species were generally sensitive to ciprofloxacin except *Morganella morganii* strains which were resistant to ciprofloxacin. The resistance to ciprofloxacin, ceftazidime, cefepime, tigercylin, colistin and fosfomycin were 65%, 40%, 23%,, 7%, 33%, 48% respectively while the prevalence of SHV, TEM and CTX genes were 42%, 63%, 35% respectively. 9.3% of the isolates had the three ESBL genes, 2.33% had *qnr*A gene, 4.65% had *qnr* B gene while none had *qnr*S gene. The most prevalent PMQR gene is *Oqx*b (25.58%) while 6.98% had the *qep* gene. *Klebsiella pneumoniae* generally had both ESBL and PMQR genes. The high prevalence of extended spectrum beta-lactamase genes in the studied strains calls for caution in the use of beta lactam antibiotics in poultry feeds. This is the first report of the occurrence of extended spectrum beta-lactamase and plasmid mediated quinolone resistance genes in *Morganella morganii* and *Leclercia adecarboxylata* strains isolated from poultry faeces.

## Introduction

The use of sub-therapeutic antibiotics as growth promoters in poultry feeds in some countries has led to increasing rate of resistance to antibiotics among pathogens in poultry environment. This is enhanced by the ability of the resistant strains to transfer acquired resistance to their progeny and other unrelated bacteria through plasmids (Apata, 2009). Resistance is common to the most frequently prescribed antibiotics such as the quinolones, beta-lactam antibiotics and aminoglycosides. This is observed in different types of infections caused by Gram-negative bacteria, especially Enterobacteriaceae, which may be difficult to treat (Schultsz & Gerlings, 2012). Of more concern are quinolones and beta-lactam antibiotics, which are commonly prescribed in human to treat different infections.

Resistance to quinolones varies across microorganisms and geographical locations. There seems to be a linkage between resistance to quinolones and the β-lactam antibiotics as ESBL genes are frequently carried on plasmids which can also carry genes encoding resistance to other antibiotics e.g., aminoglycosides thereby giving ESBL producers the characteristic broad antibiotic resistance to multiple antibiotic classes (Rawat & Nair, 2010). ESBLs are most commonly found in Enterobacteriaceae and one of the commonest ESBL producers is *Escherichia coli* (Mansouri & Ramazanzadeh, 2009). They could also occur in uncommon species of Enterobacteriaceae e.g., *Morganella morganii, Leclercia adecarboxylata* and *Citrobacter freundii. Morganella morganii* could cause nosocomial and opportunistic infections in intensive care unit patients and immunocompromised hosts (Singla et al., 2010). It has been reported to cause fatal infections in chicken (Zhao et al., 2012). *Leclercia adecarboxylata* is another rare Enterobacteriaceae isolated from water, which could act as an opportunistic pathogen in immunocompromised patients. It is usually susceptible to most commonly used antibiotics including beta-lactams. However, a few cases of antibiotic-resistant *L. adecarboxylata* have been reported (Shin et al., 2012). *Citrobacter freundii* is resistant to β-lactam antibiotics due to the production of ESBL in some strains (Kanamori et al., 2011).

*Escherichia coli* and *Salmonella* spp. are often isolated in poultry and its environment as ESBL producers (Saliu, Vahjen & Zentek, 2017) while *E. coli* and *Enterobacter cloacae* were the most frequently identified organisms from hatching eggs (Mezhoud et al., 2016). There are information on ESBL-producing *E. coli* strains from poultry, but little information is available on detection of ESBL-producing *Klebsiella pneumonia, Morganella morganii, Leclercia adecarboxylata* and *Citrobacter freundii* in poultry. We have previously studied susceptibility of non *E. coli* enterobacteriaceae isolated from poultry in Ibadan, Nigeria but the strains were not identified to species level, neither were resistant genes investigated (Ayeni, Olujobi & Alabi 2015). The aim of this study was therefore to determine the presence of ESBL and PMQR resistance genes in *Klebsiella pneumonia, Morganella morganii, Leclercia adecarboxylata* and *Citrobacter freundii* strains isolated from faecal dropping of birds in 11 poultry farms from South Western Nigeria.

## Materials and Methods

### Isolation procedures

The samples were collected between September 2015 and February 2016. A total of 240 fecal droppings were collected aseptically from 11 commercial poultry farms from five states in South Western Nigeria i.e., Oyo, Ondo, Ogun, Ekiti and Osun. Immediately after collection, 1g of each fecal sample was homogenized in 9 mL of MacConkey broth (Oxoid, Cheshire, UK) supplemented with cefotaxime (2 µg/mL) and incubated at 37 °C for 24 h. The incubated broth was plated on MacConkey agar plates (Oxoid, Cheshire, UK) supplemented with cefotaxime (2 µg/mL) (Tamang et al., 2013) at appropriate dilutions and incubated at 37 °C for 24 h. All possible distinct colonies with different morphology that grew on MacConkey plates were sub cultured to get pure cultures which were stored appropriately.

### Identification of isolates

Overnight cultures of bacterial isolates were identified with MALDI-TOF apparatus (VITEK^®^ MS (Biomerieux, Nuertingen, Germany)) according to the manufacturer’s instructions. In summary, bacterial strains were grown on MacConkey agar plates for 24 h. A thin smear of the strains were made on MALDI plate and overlaid with 1 µL of matrix solution (saturated solution of α-cyano-4-hydroxycinnamic acid in 50% acetonitrile and 2.5% trifluoroacetic acid) and air dried at room temperature. The plates were put in VITEK^®^ MS (Biomerieux, Nuertingen, Germany) where the organisms were identified by comparing their mass spectra with reference spectra of the manufacturer database. Data were interpreted with scores of ≥2 considered as reliable species level identification. All identified *Klebsiella pneumonia, Morganella morganii, Leclercia adecarboxylata* and *Citrobacter freundii* were selected for further studies.

### Antibiotic susceptibility of isolates

Bacterial isolates were cultured on MacConkey agar plates and incubated overnight aerobically. Appropriate dilutions of the colonies were made and put into VITEK apparatus for MIC evaluation. The MIC values of different classes of antibiotics were determined by VITEK^®^2 compact system (AST-N248 cards; Biomerieux, Nuertingen, Germany) according to the manufacturer’s instructions.

### Detection of ESBL and PMQR genes

All the isolates were subcultured on MacConkey agar and incubated for 24 hrs. Two colonies of grown bacteria were put in an eppendorf tube containing 1 mL of molecular grade water and mixed with the aid of a vortex mixer. The mixture was boiled for 10 min in a water bath at 100 °C and afterwards centrifuged for 5 min at 1,000 rpm. The supernatant (containing the DNA) was removed carefully using a micropipette without disturbing the pellet. The DNA was stored at −20 °C for PCR analysis.

The primers and PCR conditions used in this study are shown in Table 1. The primers were synthesized by Inqaba Biotechnical Industries (Pty) Ltd, Hatfield, South Africa. Positive and negative control from our laboratory were used. All PCR mixture contained the master mix (half of the total reaction volume), primers (1% of the final volume of the supermix), molecular graded water and 1 µL of the DNA template. The final reaction volume was 20 µl. The PCR product was thereafter viewed by gel electrophoresis after staining in ethidium bromide. A band corresponding to the expected size and positive control was assessed as positive.

**Table 1:** Primers used for gene detection.

Gene	Primers	Primer sequence	Annealing temp.	Product size	References
qnrA	*Qnr(A)-F*	5-ATTTCTCACGCCAGGATTTG-3	53 °C	516 bp	Wang et al. (2009)
	*Qnr(A)-R*	5-GATCGGCAAAGGTTAGGTCA-3			
qnrB	*Qnr(B)-F*	5-GATCGTGAAAGCCAGAAAGG-3	53 °C	469 bp	Wang et al. (2009)
	*Qnr(B)-R*	5-ACGATGCCTGGTAGTTGTCC-3			
qnrS	*Qnr(S)-F*	5-ACGACATTCGTCAACTGCAA-3	53 °C	417 bp	Wang et al. (2009)
	*Qnr(S)-R*	5-TAAATTGGCACCCTGTAGGC-3			
qepA	*Qep(A)-F*	5-CTTCTCTGGATCCTGGACAT-3	53 °C	720 bp	Şahinturk et al. (2016)
	*Qep(A)-R*	5-TGAAGATGTAGACGCCGAAC-3			
oqxB	*Oqx(B)-F*	5-ATCGGTATCTTCCAGTCACC-3	56 °C	541 bp	Şahinturk et al. (2016)
	*Oqx(B)-R*	5-ACTGTTTGTAGAACTGGCCG-3			
SHV	*SHV-F*	5-TCGCCTGTGTATTATCTCCC-3	50 °C	768 bp	Maynard et al. (2003)
	*SHV-R*	5-CGCAGATAAATCACCACAATG-3			
TEM	*TEM-F*	5-GAGTATTCAACATTTTCGT-3	50 °C	857 bp	Maynard et al. (2003)
	*TEM-R*	5-ACCAATGCTTAATCAGTGA-3			
CTX-M	*CTX-F*	5-TTTGCGATGTGCAGTACCAGT AA-3	56 °C	543 bp	Edelstein et al. (2003)
	*CTX-R*	5-CGATACGTTGGTGGTGCCATA-3			

## Results

A total of 350 strains were isolated from different samples and identified. The prevalence of the studied enterobacteriaceae were 7% *Klebsiella pneumonia* (23 strains isolated from four farms), 3% *Morganella morganii* (12 strains isolated from one farm), 2% *Leclercia adecarboxylata* (seven strains isolated from two farms) and 0.3% *Citrobacter freundii.* The remaining 308 strains were strains of *E. coli* and *Enterobacter cloaceae*.

**Figure 1:**
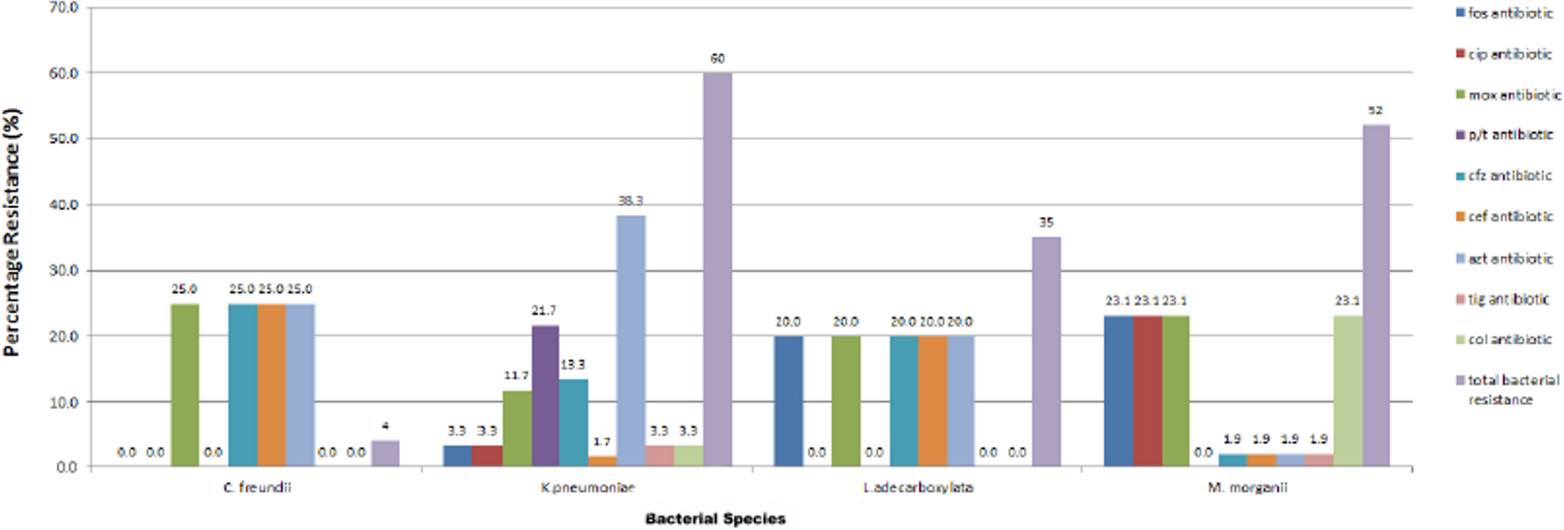
Resistance profile of bacterial strains.

All the four species were resistant to gentamycin, trimethoprim/sulphamethaxole, tobramycin, piperacillin, cefotaxime and aztreonam (except *Morganella morganii,* which were mostly susceptible to aztreonam). All the strains were sensitive to imipenem, meropenem and amikacin. Most of the strains were sensitive to tigercycline. All *Leclercia adecarboxylata* strains were resistant to moxifloxacin, ceftazidime, cefepime and fosfomycin. All *Morganella morganii* strains were resistant to colistin, fosfomycin, moxifloxacin and ciprofloxacin. The only *Citrobacter freundii* strain had additional resistance to ceftazidime and cefepime. The tested species were mostly sensitive to ciprofloxacin but all *Morganella morganii* strains were resistant to ciprofloxacin. 65% of the isolates were resistant to ciprofloxacin, 40% were resistant to ceftazidime, 23% were resistant to cefepime, 7% were resistant to tigercylin, 33% were resistant to colistin while 48% were resistant to fosfomycin (Fig. 1).

**Figure 2:**
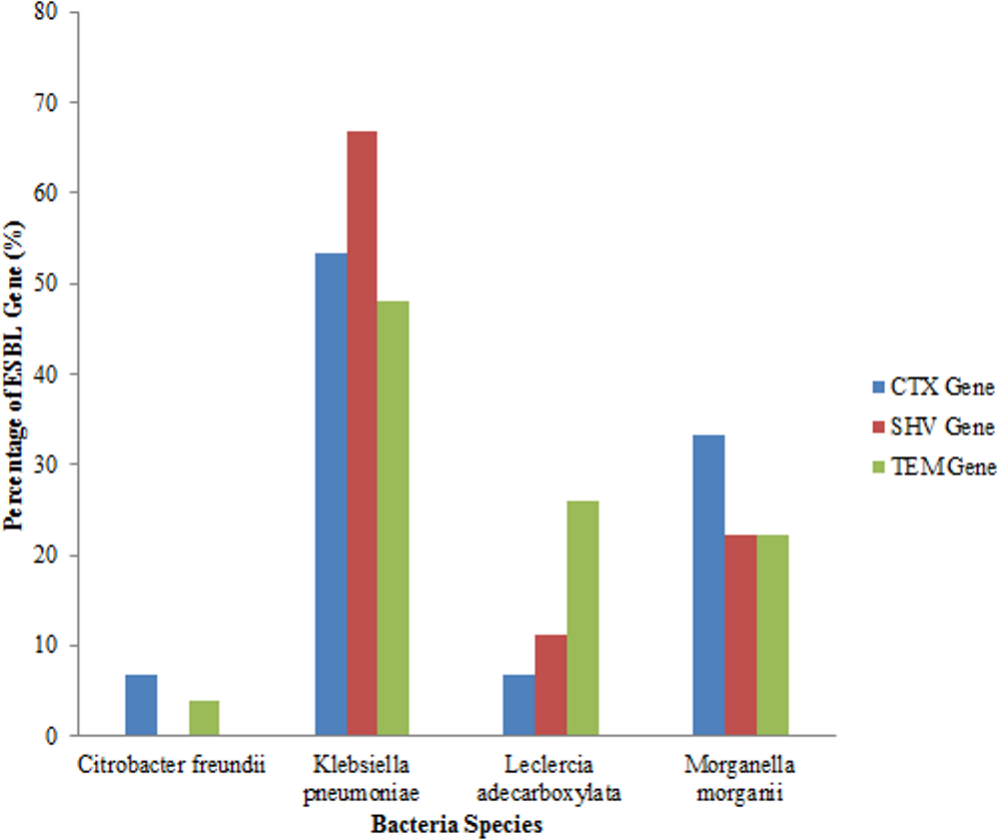
Distribution of ESBL genes in bacterial strains.

An analysis of all the isolates revealed that 18 (41.86%) had the SHV gene out of which 12 (66.67%) were *Klebsiella pneumoniae*, (four) 22.22% were *Morganella morganii* and two (11.11%) were *Leclerchia adecarboxylata.* Twenty seven (62.79%) of the total isolates had the TEM gene out of which 13 (48.14%) were *Klebsiella pneumoniae*, 7 (25.93%) were *Leclerchia adecarboxylata,* six (22.22%) were *Morganella morganii* and 1 strain of *Citrobacter freundii.* Also, 15 (34.88%) of the total isolates had the CTX-M gene with 8 (53.33%) being *Klebsiella pneumoniae,* one (6.67%) was *Leclerchia adecarboxylata,* 5 (33.33%) were *Morganella morganii* and 6.67% was one isolate of *Citrobacter freundii.* Fifteen (34.88%) isolates had both the SHV and TEM genes while four (9.3%) isolates had the three genes and seven (16.28%) isolates had none of the genes. All the *Leclerchia adecarboxylata* species had the SHV gene while *Klebsiella pneumoniae* had more SHV and TEM genes than CTX-M gene (Fig. 2).

**Figure 3:**
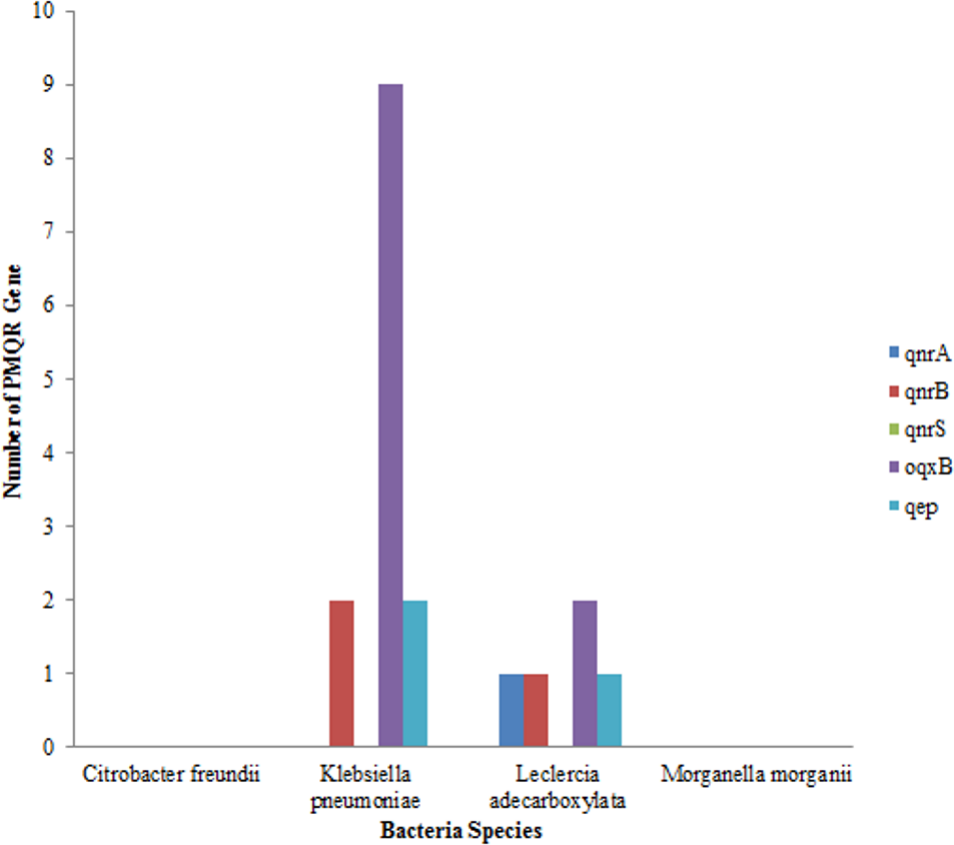
Distributions of PMQR among bacterial strains.

Out of the total of 43 isolates, *qnr* A gene was only detected in one isolate of *Leclerchia adecarboxylata*, only two isolates of *Klebsiella pneumoniae* had the *qnr* B, gene while no isolate had the *qnr* S gene. The most prevalent PMQR gene was *Oqxb* in 11 strains (nine isolates of *Klebsiella pneumoniae* and two isolates of *Leclerchia adecarboxylata*). In addition, two isolates of *Klebsiella pneumoniae* and one isolate of *Leclerchia adecarboxylata* had the *qep* gene. None of the isolates had all the PMQR genes (Fig. 3).

## Discussion

Antibiotic resistance has become a menace in the environment, as it has rendered the use of several antibiotics in the treatment of certain infections ineffective. A high prevalence of resistant bacteria genes in poultry environment may increase the risk of spread to humans, as the poultry environment is constantly in contact with humans. Observed total resistance of all studied strains to cefotaxime, piperacillin, tobramycin, trimethoprim/sulfamethoxazole and gentamicin conforms to the study by Bradford (2001), in which it was reported that β-lactam antibiotics such as ceftazidime, cefotaxime and oxyimino-monobactam were ineffective against studied bacteria however, carbapenems and cephamycine were effective against ESBL producing strains (Seija et al., 2015). This was also observed in this study, with total susceptibility of all tested organisms to amikacin, imipenem and meropenem. Carbapenems are effective in the treatment of infections caused by multi-drug resistant Gram-negative bacteria including those that produce ESBLs (Walsh et al., 2005). All *Morganella morganii* and *Leclercia adecarboxylata* strains in this study were resistant to fosfomycin. It has also been previously noted that ESBL producing isolates displayed higher fosfomycin resistance than ESBL negative strains (Seija et al., 2015). The total resistance of *Morganella morganii* strains to fosfomycin, colistin and ciprofloxacin is alarming. Demir & Buyukguclu (2013) has also previously reported resistance of all *Morganella morganii* strains from urogenital infections to fosfomycin and Arca, Reguera & Hardisson (1997) has also reported incorporation of alpha-glycerolphosphate in a fosfomycin resistant *Morganella morgannii* strain. However, Seija et al. (2015) have successfully reported treatment of multidrug resistant *Morganella morganii* in sepsis with fosfomycin thereby suggesting that the resistance is not intrinsic. Resistance of *Morganella morganii* to colistin has been described as intrinsic (Liu et al., 2016).

TEM gene was the most prevalent ESBL gene detected followed by SHV and CTX-M gene in clinical isolates (Machado et al., 2007; Sedighi et al., 2017). Fortini et al. (2011) has also reported dominance of bla (TEM) gene in Enterobacteriaceae isolated from Nigeria and only one strain was positive for the CTX-M gene. However, more prevalence of SHV gene has been reported in isolates from humans in Brazil (Dropa et al., 2009; Naim, Saeed & Malik, 2018). Prevalence of 96.7% ESBL has been previously reported in *Klebsiella pneumonia* isolated from commercial broiler slaughter plant in Shandong province of China (Wu et al., 2016). This is comparable with the results obtained in this study.

There seems to be a linkage between resistance to quinolones and the β-lactam antibiotics. It has been demonstrated that fluoroquinolone resistance can be mediated by co-transfer of the *qnr* determinant on ESBL-carrying plasmids (Wang et al., 2004) In some enterobacteriaceae, quinolone resistance is more frequent in ESBL positive stains than in ESBL-negative strains which may be due to combined effects of several mechanisms of resistance (Martinez-Martinez et al., 2002). Cremets et al. (2011) also noted that a CTX-M-15 producing isolate was highly resistant to fluoroquinolones and harboured mutations in the QRDR with two PMQR determinants.

*Klebsiella pneumoniae* strains had the highest level of resistance to antibiotics, which can be related to the presence of both ESBL and PMQR resistance genes. The most prevalent PMQR gene is *oqx*B with more abundance in *Klebsiella pneumonia* strains. (Yuan et al., 2012) and Szabó et al. (2018) also reported the presence of *oqx*AB gene in all human *K. pneumoniae* strains investigated. However, in a recent study on *Klebsiella* strains isolated from broiler, *qnr*B was the most dominant followed by *qnr*S and with low prevalence of *qep*A and *qnr*A (Wu et al., 2016).

In *Morganella morganii*, the prevalence of the three ESBL genes was relatively high with the TEM gene being the most prevalent. Al-Muhanna, Al-Muhanna & Alzuhari (2016) also showed that all *Morganella morganii* isolates tested for the ESBL had all the genes. *Morganella morganii* produces an inducible, chromosomally encoded AmpC β-lactamse, which can be implicated for its natural resistance to aminoopenicillins, amoxicillin-clavulanate, first and second generation cephalosporins with aztreonam, carbenicillin, and tazobactam being effective transient inactivators of some variants (Power et al., 2006). This is observed in this study, with most *Morganella morganii* being susceptible to aztreonam. Additionally, *Morganella morganii* is naturally resistant to tetracyclines, tigectycline, polymyxins and nitrofurantoin (Leclercq et al., 2013). However, intermediate resistance to tigercycline was observed in this study. Although high resistance to ciprofloxacin was observed in *Morganella morganii* in this study, they didn’t possess any of the PMQR genes investigated. The observed resistance may be due to an univestigated gene. Resistance of *Morganella morganii* to quinolones has been linked to *qnr*D, two new *gyr*B mutations (S463A, S464Y) and one *par*C mutation (S80I) (Nasri et al., 2014; Szabó et al., 2018).

All the *Leclercia adecarboxylata* species had the TEM gene, which may be responsible for their complete resistance to ceftazidime and cefotaxime. There was a moderate prevalence of SHV and CTX-M genes. Garcia-Fulgueiras et al. (2014) also reported the first presence of SHV and TEM genes in *Leclercia adecarboxylata*. The first case of CTX-M in *Leclercia adecarboxylata* was reported in a multi-drug resistant strain harbouring CTX-M and TEM gene (Shin et al., 2012). The prevalence of *qep* and *oqxB* gene was low in *Leclercia adecarboxylata*. In addition, three *Morganella morganii* and one *Leclercia adecarboxylata* had all three (SHV, TEM and CTX-M) genes; this increases the probability of these organisms developing resistance to quinolones.

### Limitation of the study

The observed resistance to ciprofloxacin in *Morganella morganii* strains could be due to *qnr* D gene. However, the gene was not investigated in this study.

## Conclusion

This study reports high prevalence of *oqx*B, TEM and SHV in poultry faecal dropping. To the best of our knowledge, this is the first study reporting occurrence of ESBL and PMQR in *Morganella morganii* and *Leclercia adecarboxylata* isolated from poultry.

##  Supplemental Information

10.7717/peerj.5053/supp-1 Data S1 Raw data

10.7717/peerj.5053/supp-2 Data S2 Supplementary data

## Additional Information and Declarations

### Data Availability

The following information was supplied regarding data availability: The raw data are provided in the Supplemental Files.
